# Depressive disorders after wishplash cervical spine injuries in young females

**DOI:** 10.1192/j.eurpsy.2021.2227

**Published:** 2021-08-13

**Authors:** N. Syrmos

**Affiliations:** Aristotle University Of Thessaloniki, Thessaloniki, Greece

**Keywords:** Depressive disorders, wishplash cervical spine injuries, females

## Abstract

**Introduction:**

Wishplash cervical spine injuries are serious traumatic situations

**Objectives:**

Aim of this study is to present cases of deppresive disorders after wishplash cervical spine injuries in young females

**Methods:**

4 cases are presented. Range of age between 20 and 30 years old. All of them reported depressive disorders during the post traumatic period after wishplash cervical spine injuries mainly due to road traffic accidents.

**Results:**

All of them they receive appropriate neurological, psychiatric, psycological and rehabilitation support and treatment. They managed to have a good outcome after 12 months follow up.

**Conclusions:**

The development of depressive disorders after such traumatic events remains a strong predictor of a variety of difunctions (social, personal, work etc). The emergence of depressive disorders in many cases remains unexplored and poorly understood. The effect into the the overall health remains a very important factor to investigate. The combination and collaboration of the various medical disciplines is essential in order to help young people.

**Disclosure:**

No significant relationships.

